# Study of the inhibitory effects of chrysin and its nanoparticles on mitochondrial complex II subunit activities in normal mouse liver and human fibroblasts

**DOI:** 10.1186/s43141-021-00286-0

**Published:** 2022-01-28

**Authors:** Eman M. Ragab, Doaa M. El Gamal, Tarek M. Mohamed, Abeer A. Khamis

**Affiliations:** Biochemistry Division, Chemistry Department, Faculty of Science, Tanta University, Tanta, 31527 Egypt

**Keywords:** Succinate dehydrogenase, Chrysin, Chitosan, Nanoparticles, Mitochondria, Complex ΙΙ

## Abstract

**Background:**

Mitochondrial complex ΙΙ has a unique biological role owing to its participation in both the citric acid cycle and the electron transport chain. Our goal was to evaluate the succinate dehydrogenase and ubiquinone oxidoreductase activity of mitochondrial complex II in the presence of chrysin and chrysin–chitosan nanoparticles. Chrysin chitosan nanoparticles were synthesized and characterized using ultraviolet spectroscopy, Fourier transform-infrared spectroscopy, X-ray diffraction, transmission electron microscopy, scanning electron microscopy, drug release, and zeta potential. The binding affinity of chrysin to complex II subunits was assessed by molecular docking. The IC_50_ values were measured in a suspension of mouse mitochondria, and the inhibitory effect of chrysin and chrysin chitosan nanoparticles on mitochondrial complex ΙΙ was determined.

**Results:**

The free energy of binding between chrysin and complex ΙΙ subunits A, B, C, and D was −4.9, −5, −8.2, and −8.4 kcal/mol, respectively. The characteristic peak of chrysin was confirmed at 348 nm. The chrysin chitosan nanoparticles contained characteristic bands of both chrysin and chitosan. The crystalline nature of chrysin chitosan nanoparticles was confirmed by X-ray powder diffraction measurements showing the characteristic Bragg peaks of (11.2°), (32.2°), (19.6°), (27.6°), and (31.96°). Transmission and scanning electron microscopy revealed their spherical shape and an average particle size of 49.7 ± 3.02 nm. Chrysin chitosan nanoparticles showed a burst release within the initial 2 h followed by a steady release at 8 h. Their zeta potential was positive, between +35.5 and +80 mV. The IC_50_ of chrysin, chitosan nanoparticles, chrysin chitosan nanoparticles, and 5-fluorouracil was 34.66, 184.1, 12.2, and 0.05 μg/mL, respectively, in adult mice liver and 129, 311, 156, and 8.07 μg/mL, respectively, in normal human fibroblasts. When comparing the inhibitory effects on complex ΙΙ activity, application of the IC_50_ of chrysin, chitosan nanoparticles, chrysin chitosan nanoparticles, and 5-fluorouracil resulted in 40.14%, 90.9%, 86.7%, and 89% decreases in SDH activity and 70.09%, 86.74%, 60.8%, and 80.23% decreases in ubiquinone oxidoreductase activity in normal adult mice, but 80.9%, 89.06%, and 90% significant decreases in SDH activity, and 90%, 85%, and 95% decreases in ubiquinone reductase after treatment with chrysin, chrysin chitosan nanoparticles, and 5-fluorouracil, in normal human fibroblasts, respectively.

**Conclusions:**

Chrysin and CCNPs exhibit potent inhibitory effects on SDH activity ubiquinone oxidoreductase activity.

**Supplementary Information:**

The online version contains supplementary material available at 10.1186/s43141-021-00286-0.

## Background

Mitochondria are organelles known for their function in cellular respiration, specifically for connecting molecular oxygen (O_2_) reduction to ATP synthesis [1]. The electron transport chain (ETC) in mitochondria regulates the key metabolic pathway of oxidative phosphorylation (OXPHOS), which is one of mechanisms of energy homeostasis mechanisms [2]. OXPHOS is defined as the metabolic activity by which cells convert energy in the inner membrane of mitochondria via five functional enzymatic complexes—complex I (NADH: ubiquinone oxidoreductase), complex II (succinate: ubiquinone oxidoreductase), complex III (ubiquinol: cytochrome c oxidoreductase), complex IV (cytochrome c oxidase), and complex V (ATP synthase)—to oxidize nutrients, eventually creating molecular oxygen and releasing ATP [3]. OXPHOS begins with the entrance of electrons into complex I of the respiratory chain, which are subsequently passed to the following complex by sequential transfer, eventually resulting in ATP creation [4].

Succinate dehydrogenase (SDH), also known as mitochondrial complex II, is a mitochondrial enzyme that participates in both the citric acid cycle and the ETC, oxidizing succinate to fumarate and reducing ubiquinone to ubiquinol [5]. SDHA, SDHB, SDHC, SDHD, SDHAF1, and SDHAF2 are the six subunits that form SDH, the last two of these code for associated accessory factors [6]. SDH has previously been directed to the ubiquinone binding site to inhibit the ETC and production of ATP and ROS, as well as to induce apoptosis.

Natural components found in food and medicinal plants are considered an important resource for the discovery of new and important therapeutic molecules. Biomolecules are highly diverse, with potent antioxidant, anti-inflammatory, and anticancer properties. As shown by quantitative structure-activity relationship studies, biomolecules can be used as templates for chemical modifications to improve the efficiency, safety, and bioavailability of compounds [7]. Chrysin is a naturally occurring flavone that is abundant in various plant extracts, including propolis and honey [8]; it has emerged as an outstanding health-beneficial compound owing to its anti-inflammatory, antioxidant, antidiabetic, antiallergic, antibacterial, antiestrogenic, and anticancer capabilities [9].

Given the rapid growth of technology, it appears that there are no limitations to the possibilities through which nanotechnology can be used to serve and advance the human race. Nanotechnology is a rapidly developing field of technology worldwide that has caught the attention of both scientists and clinicians. Nanoparticles (NPs) are typically used for a wide range of applications. Most of those applications make use of the high surface area to mass ratio of NPs, which provides a large functional surface for the binding, adsorption, and carrying of other compounds [10].

We used molecular docking to investigate the binding affinity of chrysin with SDH and revealed its binding characteristics. We encapsulated chrysin within the natural polymer chitosan, which exhibits adhesiveness, biocompatibility, and biodegradability, and is widely used for biological and biomedical applications. The positively charged chitosan molecule, which facilitates adherence to negatively charged cell membranes and enhances drug delivery, contributed to the improved stability of chrysin–chitosan nanoparticles (CCNPs) [11, 12]. This work aimed to study the effect of chrysin and CCNPs, compared that of with 5-fluorouracil, on the activity of mitochondrial complex II (succinate: ubiquinone oxidoreductase), both in theory using molecular docking and experimentally using normal adult mice and normal human fibroblasts.

## Methods

### Chemicals

Chrysin (>99%), Tris HCl, 5-fluorouracil (>99%), and CS (M_w_ = 100–300 kDa, 70–75% deacylated). Trisodium pentapolyphosphate (TPP, 99%), Tween 80, and mitochondria isolation buffer (mannitol (>95%), 4-(2-hydroxyethyl) piperazine-1-ethane sulfonic acid, *N*-(2-hydroxyethyl), and piperazine-*N*-(2-ethane sulfonic acid) [HEPES]), and MTT [3–(4,5-dimethylthiazol-2-yl)-2,5-diphenyltetrazolium bromide]. All these chemicals were of analytical grade and purchased from Sigma-Aldrich Company in Germany. Dulbecco’s modified Eagle’s medium (DMEM) and fetal bovine serum (FBS) were purchased from (GIBCO, New York, USA). L-Glutamine, trypsin-EDTA, and trypan blue were purchased from Invitrogen (VIC, Australia). Penicillin/streptomycin was purchased from Thermo Fisher Scientific (Waltham, MA, USA).

### In silico studies

The protein structure of SDH was retrieved and processed for in silico molecular docking investigations using the RCSB Protein Data Bank (PDB) (www.rcsb.org). The PDB contains data on three-dimensional structures of biomacromolecules. For SDH, a grid box was created around the co-crystallized ligand binding site [13]. The experimentation on molecular docking was performed using Docking Server 2009 and Discovery Studio Software Tools 2019 [14].

### Synthesis of chrysin chitosan nanoparticles (CCNPs)

After pure chrysin powder was dissolved in 96% methanol, the solution was combined with 0.1% w/v chitosan solution in 0.1% acetic acid under magnetic stirring. Then, 40 mL TPP solution (0.1% w/v) and 1.075 mL Tween-80 solution (0.01% v/v) were added dropwise to the solution under magnetic stirring at 1000 rpm to produce chrysin-loaded chitosan nanoparticles [15].

#### Physical characterization of CCNPs

The ultraviolet (UV) absorption of the supernatant of CCNPs subjected to ultracentrifugation was used to the measure encapsulation efficiency (EE) of chrysin. By analysis of the supernatants of standard chrysin solutions (10–100 g/mL) in a UV/Vis spectrophotometer (Shimadzu, Japan), the relevant calibration curves were created. Measurements of chrysin were performed at 348 nm (λ_max_) [16].

The encapsulation efficiency (EE) of chrysin was calculated from the following formula:

1 $$\mathrm{EE}\ \left(\%\right)=\left(\frac{\ \mathrm{Amount}\ \mathrm{of}\ \mathrm{chrysin}\ \mathrm{encapsulated}\ \mathrm{in}\ \mathrm{NPs}}{\mathrm{Initial}\ \mathrm{amount}\ \mathrm{of}\ \mathrm{chrysin}\ \mathrm{used}}\right)\times 100$$

All measurements were performed in triplicate and the results were reported as the mean ± standard deviation [17].

#### In vitro release study

For the in vitro release study, CCNPs (0.1 g) were placed in 1× PBS, pH 7.4 (50 mL). The complete release system was maintained at 37°C under 100 rpm continuous stirring. Every 2 h for 24 h, 1 mL of solution was removed and replaced with 1 mL of buffer [15]. The amount of loaded chrysin was analyzed by UV spectrophotometry. The amount of chrysin released from microspheres over a given time was determined via the following equation: Chrysin release (%) = amount of chrysin released from microspheres/total loading amount of chrysin in microspheres × 100 [15].

#### UV analysis

A Shimadzu (UV-3101 PC) spectrometer attached to a shaking water bath (Julabo SW20C) was used to measure electronic absorption spectra of chrysin [18].

#### Fourier transform infrared spectroscopy (FTIR) analysis

The molecular bonding formation between chrysin and chitosan nanoparticles was studied using the infrared (IR) spectra recorded on KBr disks using a Perkin Elmer 1720 spectrophotometer in the range of 4000–400 cm^−1^. Pure chitosan nanoparticles and chitosan nanoparticles loaded with chrysin were measured [19].

#### X-ray powder diffraction (XRD) spectroscopy

A Philips X PERT-PRO X-ray diffractometer (GNR, APD2000PRO, Italy) equipped with a monochromatic Cu K (1.5406 A) x-ray source was used to produce the XRD patterns of chitosan and CCNPs. The voltage employed was 45 kV and the angle range was scanned from 5 to 35° with a step size of 0.0167° [20].

#### Analysis of morphology and particle size of nanoparticles

The morphological features of the CCNPs were investigated using a JEOL JEM-2100 high-resolution TEM operated at an accelerating voltage of 200 keV. Gatan Digital Micrograph software was used to view, collect, and interpret the TEM images. A dilute suspension of CCNPs in ethanol was dropped onto copper grids to prepare the samples for TEM examination [21, 22]. SEM was performed to determine the morphology, size, and shape of the surface of the particles (TESCAN VEGA 3 SBH model). The sample was placed in an ion sputter on an electron microscope of a metal stub and coated in gold. A random scan of the stub was taken for the CCNPs [23].

#### Determining zeta potential

The electrophoretic mobility (UE) of CCNPs was measured using a folded capillary cell and the zeta potential (**ζ**) was measured using a Zetasizer Nano Series (Brookhaven, USA). For the experiment, 1 mL of diluted CCNPs was used. All measurements were performed in triplicate [24].

### Isolation of mitochondria from normal adult mice

Five adult male Swiss albino mice, weighing 28–30 g, were purchased from the Faculty of Pharmacy, Alexandria University, Egypt. The mice were maintained under suitable temperature conditions and a 12-h light/dark cycle. The experimental animals were kept in accordance with the National Institute of Health’s Guidelines for the Care and Use of Laboratory Animals. The experiments on animals were monitored by the Egyptian Ethical Committee of Tanta University’s Faculty of Science (IACUC-SCI-TU-0165) [25]. At the end of the experimental period, the mice were euthanized by cervical dislocation after intraperitoneal (*i.p.*) administration of sodium pentobarbital anesthesia (300 mg/kg) [26]. The liver was removed from the peritoneal cavity. The gallbladder was then located and removed using a scalpel as previously described [27, 28].

### Determining IC_50_ of chrysin and CCNPs on succinate-ubiquinone oxidoreductase activity (complex II) in normal adult mice

The MTT technique was used to determine the activity of succinate dehydrogenase and to determine IC_50_ by examining the inhibitory effect of different concentrations of chrysin (5–100 μM), CCNPs (5–160 μM), CNPs (200–2000 μg/mL), and 5-fluorouracil (5–40 μM) on the substrate of succinate (400 mM), with rotenone (40 mg/mL), antimycin A (2 mg/mL), and KCN (40 mM) added into the mitochondrial suspension (50 μL) [29].

### Determination of protein content

The mitochondrial protein content was detected using the Bradford assay [30].

### Determining the effect of the IC_50_ values of chrysin, CCNPs, CNPs, and 5-fluorouracil on succinate dehydrogenase and coenzyme Q reductase activities (complex II)

#### Effect of IC_50_ values of chrysin, CCNPs, 5-fluorouracil on SDH activity by the MTT test in normal adult mice

The MTT test was used to determine succinate dehydrogenase activity. Briefly, the mitochondrial suspension (50 μL) was incubated with the IC_50_ concentrations of chrysin (34.66 μg/mL), CNPs (184.1 μg/mL), CCNPs (12.2 μg/mL), and 5-fluorouracil (0.05 μg/mL) in normal cell lines; then, succinate (400 mM) was added as a substrate and rotenone (40 mg/mL), antimycin A (2 mg/mL), and KCN (40 mM) were added as inhibitors of complex Ι, ΙΙΙ, and ΙV of the ETC, respectively. The reaction volume was made up to 1 mL with 50 mM phosphate buffer and then incubated at 37°C for 30 min. Then, 50 μL of MTT (0.025%) was added to the mitochondrial suspension. In the next step, 50 μL of dimethyl sulfoxide (DMSO) was used to dissolve the formed formazan crystals. Finally, the absorbance at 570 nm was assayed using an ELISA reader (Tecan, Rainbow Thermo, Austria) [29].

#### Determination of succinate dehydrogenase–coenzyme Q oxidoreductase activity (CII)

The mitochondrial suspension (50 μL) obtained from normal adult mice was incubated with the IC_50_ values of chrysin, CNPs, CCNPs, and 5-fluorouracil, and the specific activity was detected as described in recent methods [31, 32]. The activity of complex ΙΙ was expressed as nmol/min/mg protein using the standard DCPI curve [33].

### In vitro cell viability studies using the MTT assay

#### Cell culture

Frozen human normal fibroblast cells (ATCC-CRL-2524) were cultured in accordance with standard cell culture protocols. The cells were cultured in DMEM containing 4.5 g/L glucose and 2% L-glutamine and supplemented with 10% FBS and 1% penicillin-streptomycin solution. Initially, the cells were seeded at low density and maintained at 37°C in a 5% CO_2_ incubator with 95% humidity.

#### Cell viability using MTT assay

The MTT tests were used to assess cell proliferation (viability) in accordance with the method of Denizot and Lang [34]. The cells were counted and then re-seeded in 96-well plates to a final concentration 1 × 10^5^ cells/mL (3 × 10^4^ cells/well). The cells were treated with different concentrations of chrysin (12.5–100 μM), CNPs (500–2000 μg/mL), CCNPs (12.5–100 μM), and 5-fluorouracil as a parallel control (3.13–100 μM). After 48 h, the culture medium was removed and the cells were washed twice gently with ice-cold PBS, and 20 μL of MTT (5 mg/mL) was removed from the 96-well microplates. The plates were placed in a cell culture incubator at 37°C for 4 h. The microplates were then shaken at the highest speed for 20 min at room temperature; after the incubation, the medium/MTT was removed and added 100 μL of DMSO was added to each well to dissolve the formazan produced. A negative control well, containing 10 μL of MTT stock solution control added to 100 μL of medium, was used. An ELISA reader was used to determine the absorbance of the plate at 570 nm (StatFax-2100, Awareness Technology, Inc., USA) [35].

The cell viability was determined as a percentage using the following equation:

$$\% { Viability}=\mathrm{Sample}\ \mathrm{absorbance}/\mathrm{Control}\ \mathrm{absorbance}\times 100$$

IC_**50**_ = the concentration of chrysin, CCNPs, or 5-fluororacil that causes 50% inhibition of cell proliferation.

### Isolation of crude mitochondria from normal fibroblast cell lines

To isolate mitochondria from cells, 4 × 10^6^ normal fibroblasts were subjected to the protocol of Wieckowski et al. [36].

### Determination of the two activities of mitochondrial CΙΙ in normal fibroblast cell lines

#### Determination of SDH activity by MTT test with IC_50_ of chrysin, CCNPs, and 5-fluorouracil in normal cell lines

The MTT test was used to evaluate succinate dehydrogenase activity. Briefly, mitochondrial suspension (50 μL) was incubated with IC_**50**_ of chrysin (129 μg/mL), CCNPs (156 μg/mL), and 5-fluorouracil (8.07 μg/mL) in normal cell lines; then, succinate (400 mM) was added as a substrate and rotenone (40 mg/mL), antimycin A (2 mg/mL), and KCN (40 mM) were added as inhibitors of complex Ι, ΙΙΙ, and ΙV of the ETC, respectively. The reaction volume was made up to 1 mL with 50 mM phosphate buffer, then incubated at 37°C for 30 min. Then, 50 μL of MTT (0.025%) was added to the mitochondrial suspension. In the next step, 50 μL of DMSO was used to dissolve the formed formazan crystals. Finally, the absorbance at 570 nm was assayed using an ELISA reader (Tecan, Rainbow Thermo, Austria) [29].

#### Determination of succinate dehydrogenase–coenzyme Q oxidoreductase activity (CII) in normal human cell lines

The mitochondrial suspension (50 μL) obtained from normal cell lines was incubated with the IC_50_ of chrysin (129.1 μg/mL), CCNPs (1556 μg/mL), and 5-fluorouracil (8.07 μg/mL). Specific activity was detected according to the method of Taylor et al. [31]. The calculated CII activity was expressed as nmol/min/mg protein using the standard DCPI curve [33].

### Statistical analysis

The results are shown as the mean ± SEM (*n* = 3). GraphPad Prism, v.6, was used for all the statistical analyses. The tests were repeated three times and two-way ANOVA tests followed by post hoc Tukey tests were used to establish statistical significance. To assess SDH activity, we used ANOVA tests as a specialized statistical analysis test. The threshold for statistical significance was fixed at *P* < 0.05.

## Results

### In silico analysis

**Fig. 1:**
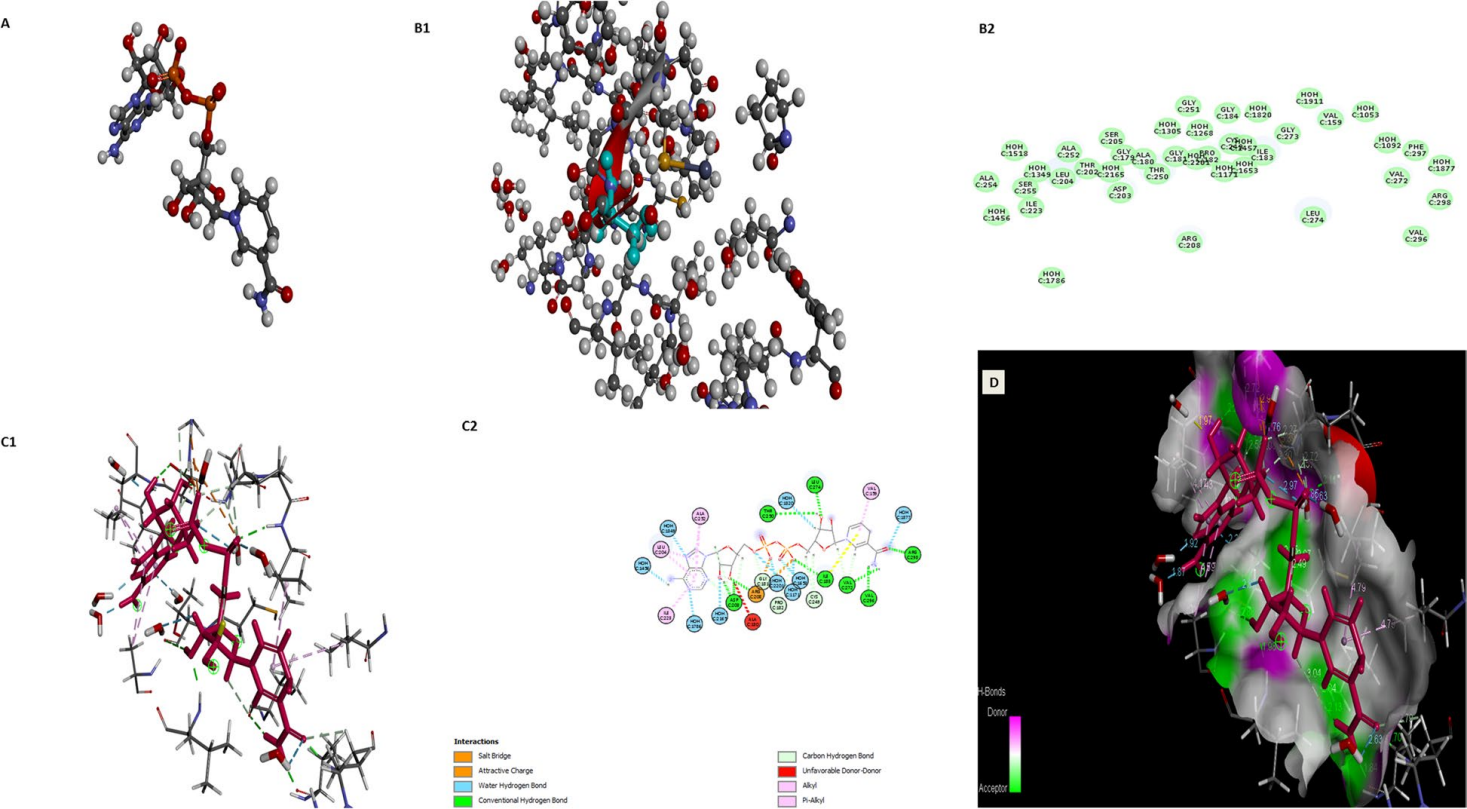
**A** Superimposition of the native ligand found within the crystal structure and the docked pose of the same ligand. **B** Active site of SDH without ligand that is represented by balls and sticks (B1) with 2D structure (B2). **C** 3D and 2D models indicate the interaction between chrysin with the active pocket of SDH-like protein’s active site. The protein is represented by molecular surface and chrysin is depicted by balls and sticks as C1 and C2 respectively. **D** 3D model binding of chrysin to the active site of SDH-like protein. Hydrogen bond interactions are indicated, with numerals indicating the distance in angstroms

Semi-empirical calculations yielded the optimal molecular structure of the inhibitor, which was used as an input file for the conformational search using a systematic search approach. We double-checked the known structures (PDB code: 1NEK, resolution 2.5) from the PDB. The natural ligand was removed from the active site and docked into the enzyme’s binding site to characterize the binding pocket of the SDH-like protein (the grid dimensions were *x* = 60 Å, *y* = 30 Å, and *z* = 10Å, with a grid spacing of 9 Å), as shown in Fig. 1A. The square deviations between the predicted conformer and the observed X-ray crystallographic conformer, 0.3 Å, were clustered together (superimposition). The active site of SDH contained 25 amino acids: GLY 251, VAL 159, GLY 179, ALA 180, GLY 181, PRO 182, IIE 183, GLY184, THR 202, ASP 203, LEU 204, SER 205, ARG 208, IIE 223, CYS 249, THR 250, ALA 252, ALA 254, SER 255, VAL 272, GLY 273, LEU 274, VAL 296, PHE 297, and ARG 298 (Fig. 1B1, B2).

Docking analysis showed that the phenolic moiety and the C–H group of chrysin were tightly bonded to the active site of SDH by several hydrogen bonds and van der Waals, hydrophobic, and electrostatic interactions. A possible conventional hydrogen bond between the (VAL272, VAL 296, ILE 183, ASP 203, ARG 298, THR 250, and LEU 274) and C–H (GLY 181, PRO 182, CYS 249) at the active site side-chain appeared to be responsible for the affinity of chrysin with SDH-like protein (Fig. 1C1, C2). The surface illustration of the minimum energy structure of the complex with chrysin docked in the active site of the SDH-like protein was determined from the molecular modeling study (Fig. 1D). The free binding energy was −4.9, −5, −8.2, and −8.4 kcal/mol.

### Physical characterization of nanoparticles

#### Encapsulation efficiency (EE; %)

Based on Eq. (1), the EE was 92.63% ± 1.30%.

#### UV analysis and in vitro release study

**Fig. 2:**
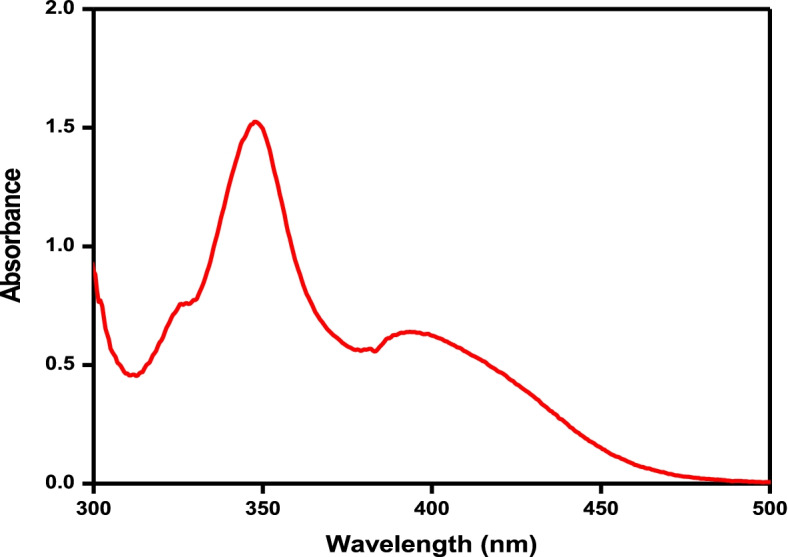
Ultraviolet absorption spectrum of chrysin

**Fig. 3:**
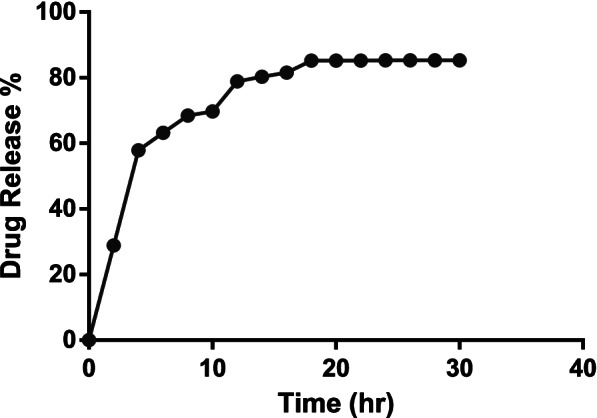
In vitro drug release profile of chrysin from the CCNPs

Chrysin showed a characteristic peak at 348 nm, as shown in Fig. 2. The in vitro chrysin release profile from CCNPs was assessed in the release medium of PBS, pH 7.4 at 37°C for stability and drug release. The chrysin release over time is shown in Fig. 3. The drug release kinetics of CCNPs showed an initial burst release followed by a steady release after 8 h. The first burst release was observed in the first 2 h with 28.86% ± 0.057% chrysin release. The second burst was observed after 8 h, with 68.43% ± 0.015% chrysin release. The graph forms a plateau from 18 to 24 h. A total of 90% ± 1.039% chrysin was released from the NPs within 18 h, as shown in Table 1.

**Table 1:** The in vitro drug release profile of chrysin from the CCNPs

NO	Time (h)	Drug release %
1	2	
2	4	28.86 ± 0.057
3	6	57.88 ± 0.0057
4	8	63.19 ± 0.0577
5	10	68.43 ± 0.0152
6	12	69.70 ± 0.035
7	14	78.9 ± 0.05
8	16	80.28 ± 0.02
9	18	81.55 ± 0.96
10	20	85.2 ± 1.039
11	22	85.2± 1.03
12	24	85.2 ± 1

#### FT-IR spectroscopy

**Fig. 4:**
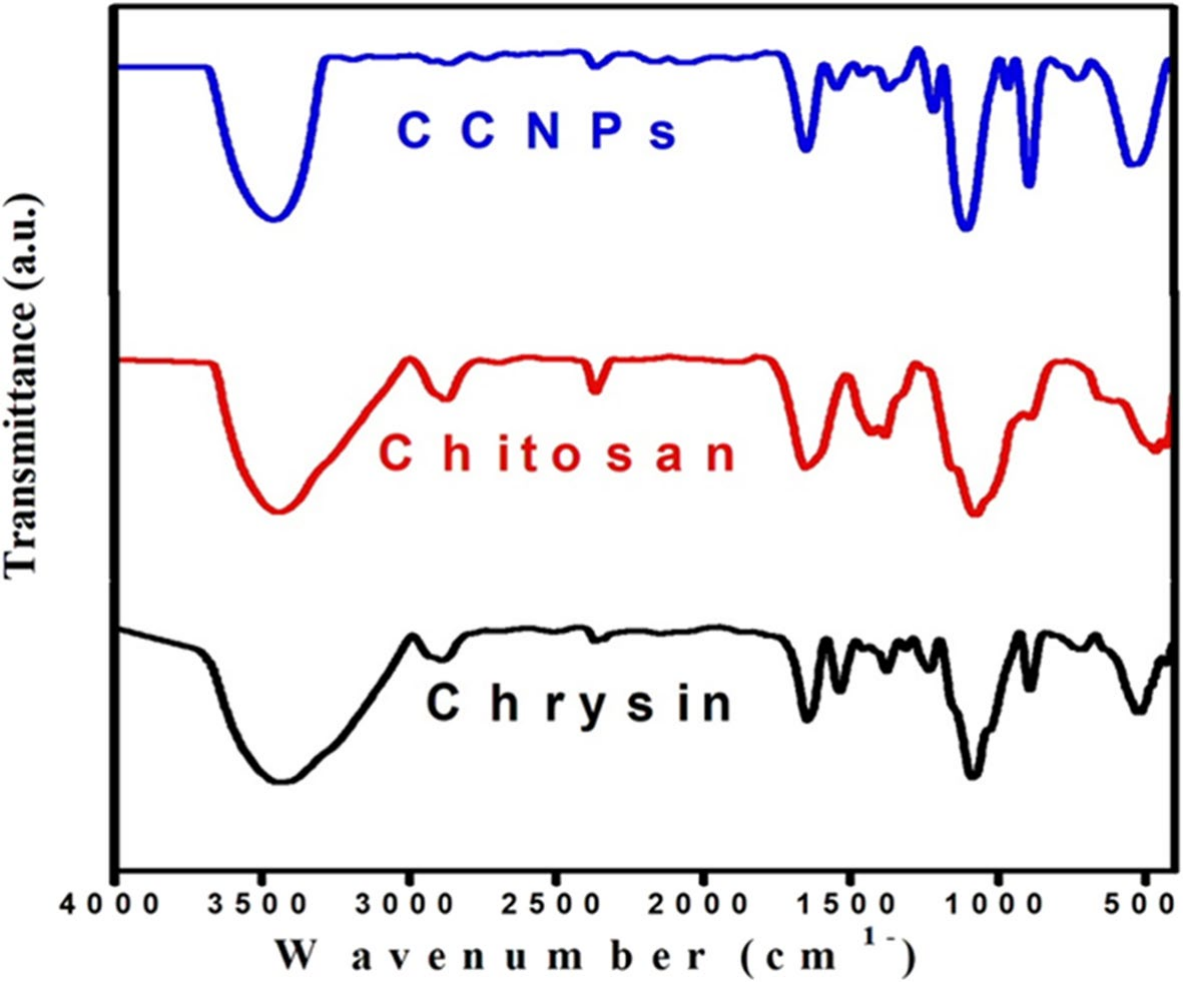
Fourier transform-infrared (FT-IR) spectroscopy

When comparing the functional groups present in CCNPs with those of their bulk counterparts, chrysin had characteristic bands at 520, 720, and 888 cm^−1^, indicating the C–C, C–O, and C–O–C stretching vibrations. The characteristic peaks at 1533 and 1653 cm^−1^ depicted the C=O group, whereas the band at 3455 cm^−1^ was due to the stretching vibration of the OH group. In contrast, chitosan results in distinct bands at 1080 cm^−1^, 1648 cm^−1^, and 2889 cm^−1^, which represent the CH_2_ group, the amide band, and the C–H stretching vibration, respectively. CCNP peaks contain characteristic bands of both chrysin (at 535 cm^−1^, 734 cm^−1^, 888 cm^−1^, 1544 cm^−1^, and 1650 cm^−1^) and chitosan (at 1648 cm^−1^ and 2391 cm^−1^). Conversely, a slight shift was also observed at 3346 cm^−1^, signifying the formation of hydrogen bonds between chrysin and the NH_2_ groups of chitosan, as shown in Fig. 4.

#### XRD patterns

**Fig. 5:**
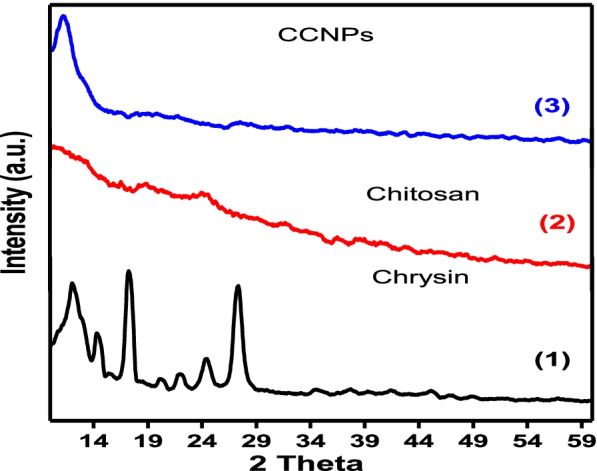
X-ray diffraction (XRD) pattern of (1) chrysin, (2) chitosan, and (3) CCNPs

The X-ray diffractogram of chrysin showed characteristic peaks at 12°, 14.4°, 17.23°, 20.18°, 22.03°, 24.64°, and 27.36° that were crystalline, whereas chitosan and TPP were semi-crystalline. For the XRD pattern of CCNPs, there was a shift in the peaks to 11.2°, 32.2°, 19.6°, 27.6°, and 31.96°, which were attributed to the interactions of chrysin/chitosan/TPP (Fig. 5).

#### Morphology and particle size of nanoparticles

**Fig. 6:**
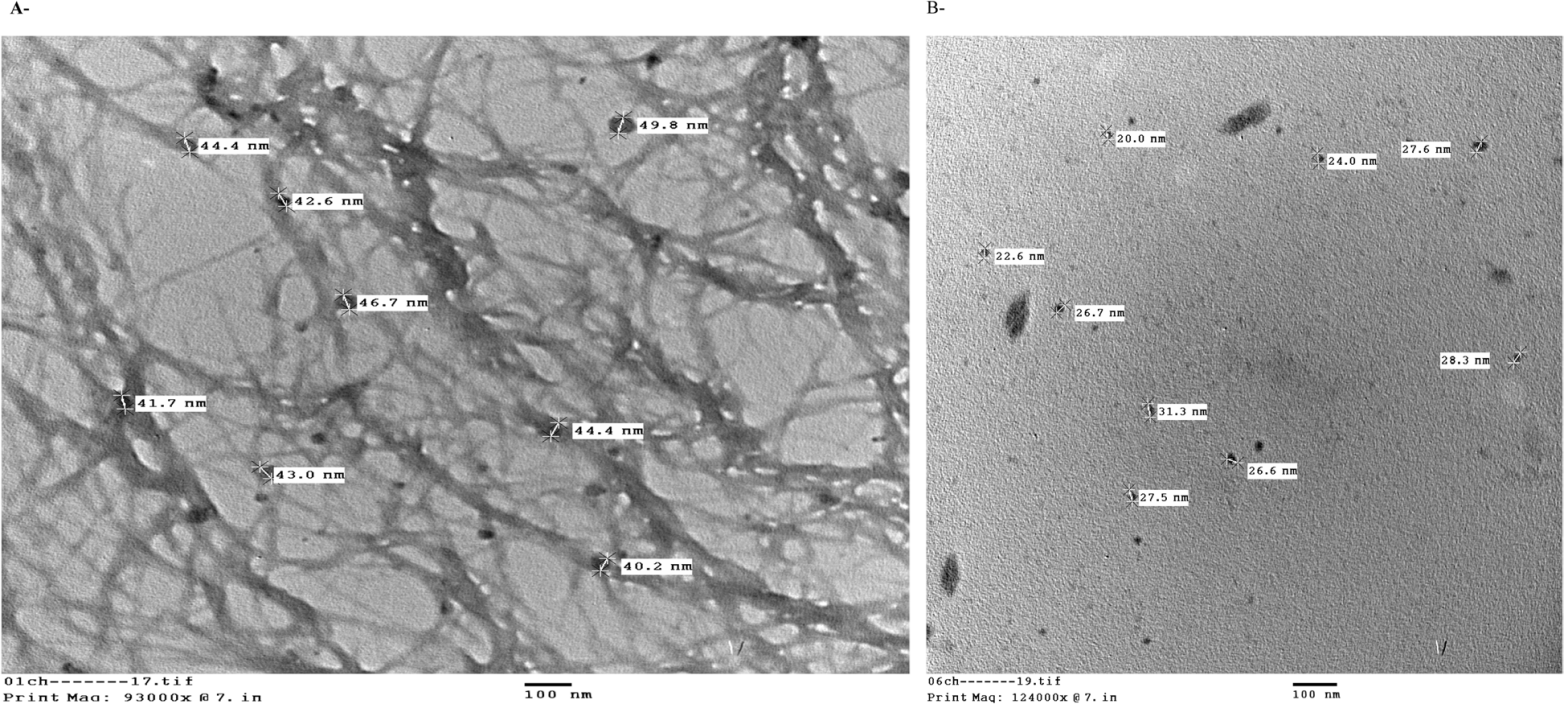
TEM micrograph showing **A** chitosan–chrysin nanoparticles (CCNPs) with average size 49.7 ± 3.02 nm. **B** Chitosan nanoparticles (CNPs) with average size 26 ± 3.3 nm

**Fig. 7:**
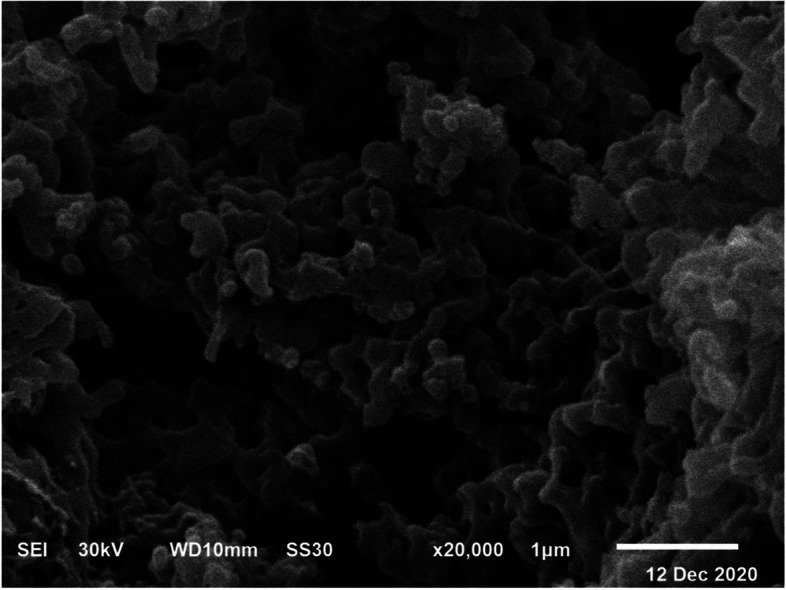
SEM micrograph showing surface morphology of chitosan–chrysin nanoparticles

TEM was used to confirm the particle size and surface morphology of CCNPs, as shown in Fig. 6. The nanoparticles were spherical with an average particle size of 49.7 ± 3.02 nm. SEM analysis was used to evaluate the size, surface morphology, and uniformity of chrysin nanoparticles (Fig. 7). The bulk of the biogenic CCNPs showed a more definite structural arrangement; they were spherical with a smooth surface and ranged from 30 to 42 nm in size. Finally, the dispersion of drug particles was substantially improved**.**

#### Zeta potential

**Fig. 8:**
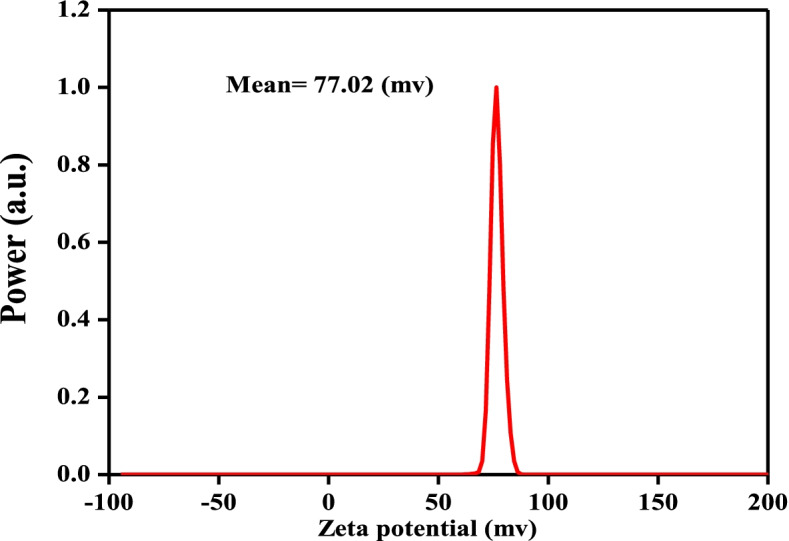
Zeta potential of chitosan–chrysin nanoparticles

Chitosan nanoparticles generally have a positive zeta potential owing to the cationic properties of the chitosan molecule. Our formulations had a positive zeta potential, of between +35.5 and +77.02 mV (Fig. 8).

### Determining IC_50_ of chrysin, CNPs, CCNPs, and 5-fluorouracil on succinate-ubiquinone oxidoreductase activity (complex II)

**Fig. 9:**
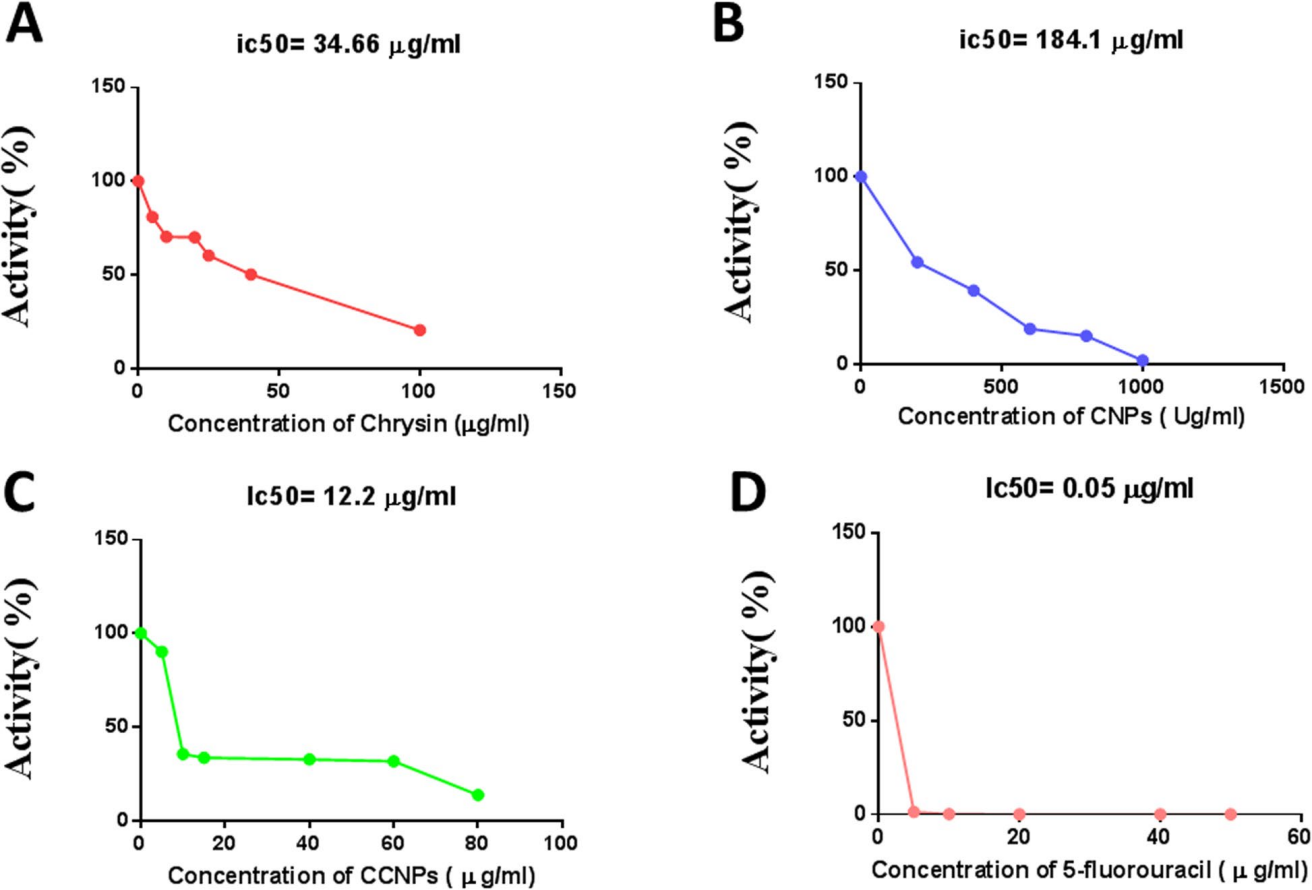
IC_50_ of **A** chrysin, **B** chitosan nanoparticles (CNPs), **C** chrysin–chitosan nanoparticles (CCNPs), and **D** 5-fluorouracil of complex ΙΙ in normal mice liver

**Fig. 10:**
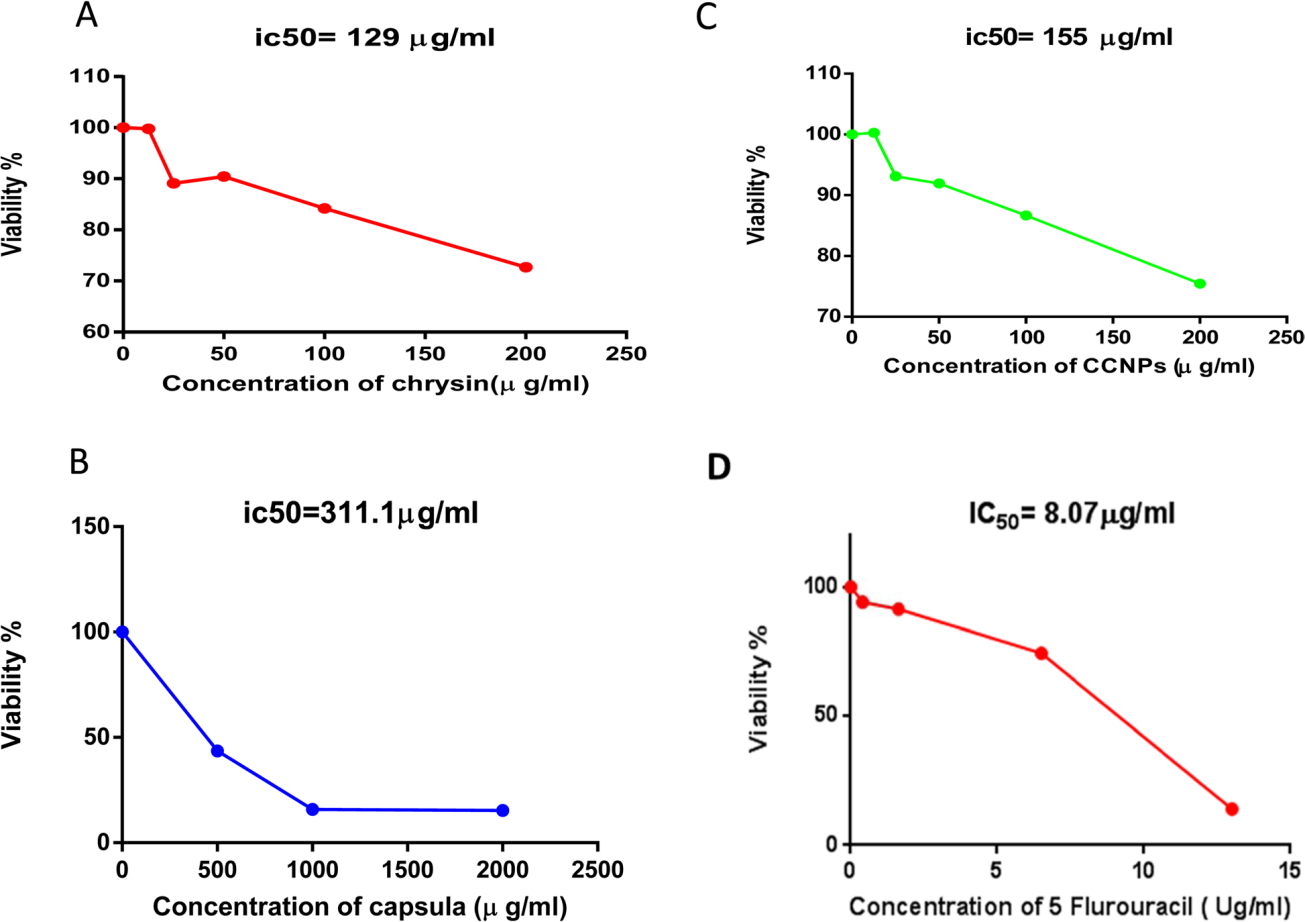
IC_50_ of compounds **A** chrysin, **B** chitosan nanoparticles (CNPs), **C** chrysin–chitosan nanoparticles (CCNPs), and **D** 5-fluorouracil of complex ΙΙ in normal fibroblast cell line

The cytotoxic effect of chrysin, CNPs, CCNPs, and 5-fluorouracil was evaluated by the MTT assay (Fig. 9), with IC_50_ values of 34.66, 184.1, 12.2, and 0.05 μg/mL in normal adult mice, and 129, 311, 156, and 8.07 μg/mL in normal fibroblasts, respectively (Fig. 10).

### Determining succinate dehydrogenase (SDH) and coenzyme Q reductase (complex II) activities by IC_50_

**Fig. 11:**
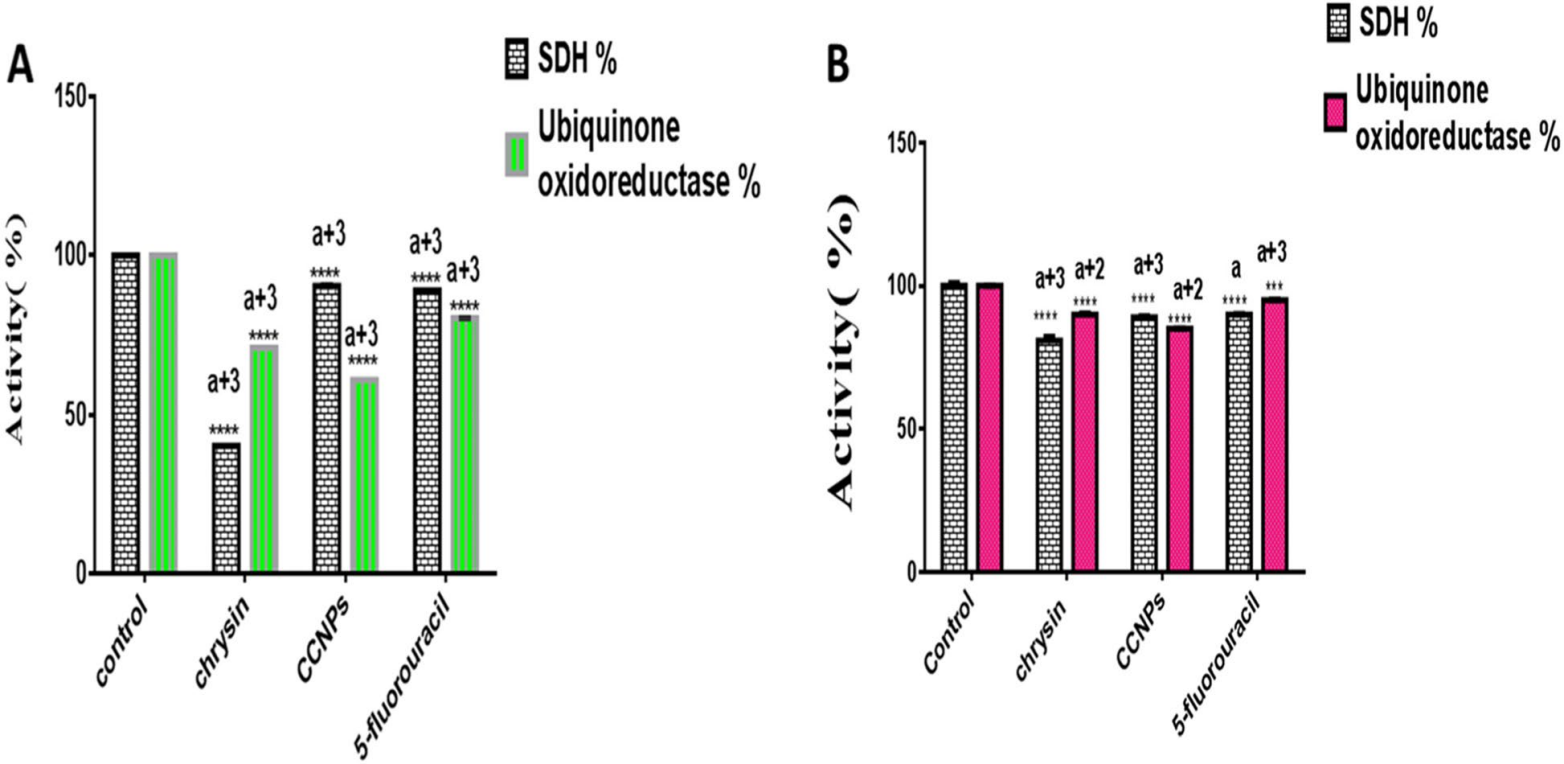
Inhibition effect of chrysin, CCNPs, and 5-fluorouracil with IC_50_ on SDH-ubiquinone oxidoreductase. Data are shown as mean ± SEM (*n* = 3). The two-way ANOVA test was carried out. **** P < 0.0001 versus the corresponding control where ^*a+3*^ *P* <0.0001, ^*a+2*^*P*< 0.001 and a value for non-significant versus each other for two activities. **A** Normal mice mitochondrial CΙΙ. (B) Normal fibroblast human cell lines

The relative SDH activity determined by the MTT test (Table 2) showed that chrysin had more potent inhibitory effects on SDH than on ubiquinone reductase. However, the relative enzyme activity of ubiquinone reductase (Table 2) showed greater targeting of CCNPs to the ubiquinone site compared with chrysin and 5-fluorouracil (Fig. 11A). In normal human fibroblasts, the relative enzyme activity of SDH, as determined by the MTT test (Table 3), showed that chrysin had more potent inhibitory effects on SDH than ubiquinone reductase. However, the relative enzyme activity of ubiquinone reductase (Table 3) showed that CCNPs affected the ubiquinone site than chrysin and 5-fluorouracil (Fig. 11B).

**Table 2:** Determination of succinate dehydrogenase (SDH) and coenzyme Q reductase (complex II) activities by IC_50_

Relative enzyme activity %	Negative control (untreated cells)	Chrysin Mean± SEM *p value	CNPs Mean± SEM *p value	CCNPs Mean± SEM *p value	5-Fluorouracil Mean± SEM *p value
Succinate dehydrogenase by MTT test	100 ± 0	40.14±0.03 *P* <0.0001	90.9± 0.005 *P* <0.0001	86.7±0.041 *P* <0.0001	89± 0.02 *P* <0.0001
Ubiquinone reductase by DCPI dye	100 ± 0	70.90±0.003 *P* <0.0001	86.74 ± 0.005 *P* <0.0001	60.8 ±0.002 *P* <0.0001	80.23 ±0.02 *P* < 0.0001

*p value versus the negative control (untreated cells)

**Table 3:** Determination of succinate dehydrogenase (SDH) and coenzyme Q reductase (complex II) activities by IC_50_ in normal fibroblast cell lines

Relative enzyme activity %	Negative control (untreated cells)	Chrysin Mean± SEM *p-value	CCNPs Mean± SEM *p value	5-Fluorouracil Mean± SEM *p value
Succinate dehydrogenase by MTT test	100 ± 0	80.90 ±0.003 *P* <0.0001	89.06 ±0.0003 *P* <0.0001	90± 0.003 *P* <0.0001
Ubiquinone reductase by DCPI dye	100 ± 0	90±0.002 *P* <0.0001	85±0.02 *P* <0.0001	95 ±0.001 *P* < 0.0001

*p value versus the negative control (untreated cells)

## Discussion

Despite the progress in modern medicine, there are numerous advantages to the use of natural compounds compared with synthetic drugs. Natural compounds have fewer side effects, are nutritionally beneficial, and are less expensive [37]. In accordance with the findings of Hamza et al. [38], chemoprotection, which is based on the use of natural food products, such as ginger, and exogenous phytochemicals to strengthen the endogenous mechanisms against various phases of cancer development, was found to be one of the most effective techniques for minimizing the impact of cancer. Their chemical constituents are believed to have better compatibility with the human body [39]. Bioactive peptides have been shown to influence carcinogenesis-related variables such as oxidative stress and inflammation. As a result, their use in the development of prospective medications, nutraceutical supplements, and other pharmaceutical items has been extensively studied [40]. Natural tablets are frequently preferred because they are less expensive, safer, and have fewer undesirable effects than conventional drugs [41]. Furthermore, a study [42] reported that natural products had enhanced antiproliferative, antidiabetic, and anti-inflammatory activities, indicating their potential use as bioactive and functional ingredients. Moreover, the study of Mu et al. [43] demonstrated that other herbal food-derived active compounds were identified by molecular docking to target the COVID-19 virus. Nanomedicine is a major field of nanotechnology that involves the nanotechnological applications utilized for diagnosis, monitoring, biological systems, and therapeutics [10].

The studies outlined in this work indicate targeting that mitochondrial trafficking appears to be a novel therapeutic strategy for this very recalcitrant disease. Respiratory complex II has increasingly been identified as a source and modulator of ROS. In previous studies by Eijkelenkamp et al. [44], it was found that the pharmacological suppression of CII, as well as its functional loss, can be used to treat a variety of illnesses, including cancer. Although the basic framework for CII involvement has been established, the finer details are still under investigation. To completely comprehend the role of CII and to investigate its therapeutic potential in cancer and other disorders, these issues must be resolved. The anticancer activities of several drugs that influence SDH activity have been investigated. One of these molecules is chrysin, an abundant natural flavonoid in beeswax; in recent studies, this molecule has been demonstrated to have many biological functions, including anti-inflammation, anticancer, and antioxidation properties. However, the mechanisms underlying the anticancer effects of chrysin are not well understood. The potential of chrysin as a chemopreventive agent has been demonstrated [45]. However, owing to its poor solubility, it is poorly absorbed in the body. Nano-encapsulation is a most interesting solution to this problem. The bioavailability of chrysin can be improved through the alteration of its pharmacokinetic properties and biodistribution. Nanoparticles made of biodegradable polymer materials, such as chitosan, have been studied as potential drugs, notably in oncology therapy. Chitosan is composed of two subunits of D-glucosamine and *N*-acetyl-D-glucosamine, which are bound together by a (1,4)-glycosidic bond [46]. The amine group on the glucosamine unit of chitosan is crucial because it provides a strong and reactive positive charge. The positive charge of chitosan can combine with an anionic molecule to produce a complex. Chitosan can also increase the number of drugs that get through the cell membrane. In our studies, we showed the effect of chrysin and CCNPs on mitochondrial CΙΙ subunits activity compared with 5-fluorouracil from both theoretical and experimental perspectives. Chrysin selected through virtual screening was docked into the active site of the target SDH subunits A, B, C, and D, by molecular docking software. Our results showed that the binding energy was −4.9, −5, −8.2, and −8.4 kcal/mol, respectively, which correlated well with the ubiquinone binding of SDH. These results strongly suggested that the SDH C and D subunits may interact with chrysin by forming a possible conventional hydrogen bond as (VAL 272, VAL 296, ILE 183, ARG 203, ASP 203, ARG 298, THR 250, LEU 274) and C–H (GLY 181, PRO 182, CYS 249) at the active site side chain. This correlated better with the ubiquinone binding subunit of SDH than the succinate and iron-sulfur cluster binding subunits [14]. This study employed the technique of ionic gelation in the manufacture of low-weight compounds with good encapsulation efficiency [47]. The physicochemical characterization of CCNPs showed a distinctive peak of UV analysis at 348 nm. The FT-IR analysis showed that the peaks of CCNPs include absorption bands for both chrysin and chitosan. However, a small shift was also noted at 3346 cm^−1^ for chitosan, resulting from the formation of chrysin and NH_2_ hydrogen bonds. In the XRD pattern of CCNPs, there was a shift in peaks that was attributed to the interaction between chrysin, chitosan, and TPP. However, the formation of CCNPs resulted in the disappearance of some chitosan peaks and the suppression of other peaks in chrysin. During blending/co-mixing with TPP, the crystalline nature of chrysin was lost, resulting in an amorphous phase. Solid-state amorphization increases compound solubility and bioavailability. Therefore, the semi-crystalline CCNPs provide an optimal structure favoring applications of tissue engineering [15, 18].

CCNPs were prepared by ionic crosslinking with TPP at a 5:1 mass ratio. Typically, the surface charge of NPs is a key parameter affecting the short- and long-term stability of NPs. In our study, the surface charge of the particles, as determined by zeta potential, was increased. The increase in surface charge could be due to less neutralization of the NH_3_ groups by the TPP crosslinker. Particles with a high positive charge are more stable and possess the properties of mucoadhesion and tissue permeation. Chitosan nanoparticles have a positive zeta potential, which improves drug delivery by facilitating attachment to negatively charged cell membranes and increases CCNPs stability [48]. It was observed that as the mass ratio increased, the size of the particle decreased. Consequently, CCNPs can more effectively supply drugs to tissue or cells. The anticancer potency can therefore be increased relative to the use of chrysin alone. The spherical form can be more easily digested by cells than the rod form of CCNPs [49]. The spherical shape was confirmed by TEM and SEM analyses, but the rough surface of the CCNPs microspheres still contained insoluble particles in the entire matrix owing to the high hydrophobicity of chrysin. Moreover, we hypothesized that the roughness of the surface remained owing to the presence of insoluble drug particles after solvent washing [20, 47].

In our studies, we evaluated the IC_50_ of chrysin, CNPs, CCNPs, and 5-fluorouracil on succinate/ubiquinone oxidoreductase activity (CII) in normal adult mice and normal human fibroblasts. The IC_50_ of chrysin, CNPs, CCNPs, and 5-fluorouracil was evaluated by MTT assay directly in crude mitochondria with inhibitors of mitochondrial complexes Ι, ΙΙΙ, and ΙV. The IC_50_ values were 34.66, 184.1, 12.2, and 0.05 μg/mL, respectively, in normal adult mice and 129, 311, 156, and 8.07 μg/mL, respectively, in normal human fibroblasts. Previous studies have shown that chitosan nanoparticles that encapsulated chrysin have weak cytotoxic effects in normal cells. Based on drug load, the IC_50_ was significantly lower in CCNPs compared with free chrysin, in addition to the nontoxic effects of CNPs [30]. These results agreed with Seydi et al. [29]. After the IC_50_ for succinate dehydrogenase (SDH) activity was determined, coenzyme Q reductase (CII) activities were evaluated. The relative enzyme activity of SDH by the MTT test showed that chrysin had more potent inhibitory effects on SDH than ubiquinone reductase. However, the relative enzyme activity of ubiquinone reductase showed that CCNPs were more targeted to the ubiquinone site than chrysin and 5-fluorouracil, as shown by their relative IC_50_ values for CΙΙ; significant decreases in SDH activity of 40.14 %, 90.92%, 86.7%, and 89%, and in ubiquinone site activities of 70.90%, 86.74%, 60.8%, and 80.23%, respectively, in normal adult mice. In contrast, in normal fibroblasts, the activities were 80.90%,89.06%, and 90% for SDH and 90%, 85%, and 95% for ubiquinone reductase by chrysin, CCNPs, and 5-fluorouracil, respectively. Based on our findings that chrysin significantly decreased the enzyme activity of SDH more than CCNPs, and CCNPs reduced ubiquinone oxidoreductase after treatment with the IC_50_, we proposed that CCNPs displayed better targeting than chrysin. Therefore, we recommend that in silico, in vitro, and in vivo studies of the inhibitory effect on mitochondria CΙΙ in most common types of cancer throughout the world should be performed.

## Conclusions

Our study showed that chrysin and CCNPs were more potent inhibitors of the SDH and ubiquinone oxidoreductase activities of CΙΙ, respectively. This has previously been proven theoretically using molecular docking, revealing the high binding affinity of chrysin for succinate-ubiquinone oxidoreductase. Further, we determined the IC_50_ values of chrysin, CNPs, CCNPs, and 5-fluorouracil against mitochondrial CΙΙ in crude mitochondria isolated from normal mouse liver using the MTT assays and in normal fibroblast cell lines. Overall, we assayed the activity of succinate-ubiquinone oxidoreductase to confirm the previous theory and reveal the mechanisms. Our results showed that chrysin significantly decreased the enzyme activity of SDH more than CCNPs, and that CCNPs reduced ubiquinone oxidoreductase following treatment at the IC_50_. Based on these inhibitory effects, we predict that chrysin and CCNPs could be considered to have therapeutic effects on mitochondrial CΙΙ in cancer cells owing to their inhibition of mitochondrial CΙΙ and ROS generation and the induction of apoptosis with the suppression of ATP generation in cancer cells. We investigated the potential anticancer effect of chrysin with respect to clinical application and the recommended evidence. We used molecular docking to provide mechanical insights into potential mechanisms of druggability.

## Supplementary Information

**Additional file 1:** The ARRIVE Essential 10: Compliance Questionnaire.

## Data Availability

Not applicable.

## Abbreviations

SDH: Succinate dehydrogenase
Chr: Chrysin
CCNPs: Chrysin–chitosan nanoparticles
TEM: Transmission electron microscopy
SEM: Scanning electron microscopy
CS: Chitosan
XRD: X-ray diffraction
ETC: Electron transport chain
OXPHOS: Oxidative phosphorylation
TPP: Trisodium Penta polyphosphate
UV: Ultraviolet
EE: Encapsulation efficiency
FTIR: Fourier transform infrared spectroscopy
UE: Electrophoretic mobility
MTT: [3–(4,5-Dimethylthiazol-2-yl)-2,5-diphenyltetrazolium bromide]
DMEM: Dulbecco’s modified Eagle’s medium
FBS: Fetal bovine serum
PDB: Protein Data Bank
